# Care of the Feet

**Published:** 1889-01

**Authors:** 


### CARE OF THE FEET.

Persons who are troubled with feet which at times emit an offensive odor, in spire of all known preventives, will be glad to learn of the following simple remedy which we have obtained from one of our eminent physicians. It is usually the case that those who are troubled in this way are subject to excessive perspiration. No amount of bathing and washing remedies the evil complained of. The softening of the skin between the toes and leakage of the fetid lymph suggest an astringent application, but even a strong solution of sulphate of zinc, will be absorbed producing swelling and more or less distress ; but oxide of zinc, not only possesses the proper astringent property, but effectually arrests the discharge, disinfecting the parts, and effecting a complete cure of the offensive malady.

